# Evaluation of the application of the Diabetes Quality of Life Questionnaire in patients with diabetes mellitus

**DOI:** 10.20945/2359-3997000000196

**Published:** 2020-03-04

**Authors:** Edilene Vieira Pereira, Fernanda Stumpf Tonin, Jaqueline Carneiro, Roberto Pontarolo, Astrid Wiens

**Affiliations:** 1 Departamento de Farmácia Universidade Federal do Paraná Curitiba PR Brasil Departamento de Farmácia , Universidade Federal do Paraná (UFPR), Curitiba , PR , Brasil

**Keywords:** Diabetes mellitus, quality of life, Diabetes Quality of Life Questionnaire

## Abstract

**Objective:**

Diabetes mellitus (DM) is a chronic disease with great impact on patients’ quality of life (QoL). This variable can be measured using reliable, standardized, and validated instruments. The purpose of this study was to evaluate the application and reporting of the Diabetes Quality of Life Measure (DQOL) or the Diabetes Quality of Life for Youths Measure (DQOLY), an adapted version for young patients with DM.

**Materials and methods:**

A systematic review of interventional and observational studies using the DQOL or DQOLY was performed. Searches were conducted in the electronic databases Medline, Scopus, Web of Science, Lilacs, and SciELO.

**Results:**

After conducting the searches, 111 studies met the inclusion criteria and were included in the qualitative analysis. Of these, 32 studies were classified as interventional and 79 as observational, with 27,481 patients. The DQOL was applied in 82 studies, the DQOLY in another 27, and two studies used both instruments. DM was classified as type 1 DM in 69 studies and type 2 DM in 35 studies. Six studies included both patients. Improvement in patients’ QoL after an intervention was observed in 13 interventional studies. Most of the studies (90%) provide a detailed description of the instrument and 52% the previous validation. The interpretation of the scores obtained varies among the studies, probably due to the differences inherent in cultural validations, translations, and adaptations.

**Conclusion:**

The application of the instruments in clinical practice must be rigorously standardized and requires an accurate understanding of psychometric and statistical concepts. Arch Endocrinol Metab. 2020;64(1):59-65

## INTRODUCTION

Diabetes mellitus (DM) is a major global epidemic and a serious public health problem ( 1 ) affecting around 415 million people worldwide ( 2 ). The chronic nature of the disease, in addition to increasing the chances of developing complications, makes DM onerous for individuals and for a country’s public health system. DM costs go beyond the direct and indirect costs of the disease to include intangible costs such as pain, anxiety, and worsening of quality of life (QoL). QoL has a great impact on the life of diabetic patients as well as a direct relationship with the maintenance of glycemic control ( 3 , 4 ).

Psychosocial and QoL assessments are fundamental health outcomes that should be measured frequently during the treatment of DM patients ( 5 ). Among the main tools for measuring QoL are the Diabetes Quality of Life Questionnaire (DQOL), developed by the multicentric group Diabetes Control and Complications Trial (DCCT) in 1988 to evaluate the effects of intensive treatment on the QoL of patients with type 1 DM (T1DM). The instrument was validated for use in patients with type 2 DM (T2DM), and the version for adolescents and young adults, the Diabetes Quality of Life for Youths Measure (DQOLY), was adapted by Ingersoll and Marrero in 1991 ( 6 ). The questions of these instruments are based on three perspectives: the impact generated by DM, satisfaction, and concern about the effects of the disease ( 7 ). The instruments are not interchangeable between adults and adolescents, since in youths mental health seems to contribute more to the perception of QoL than to physical health ( 8 ). In Brazil the DQOL and DQOLY tools were validated in 2008 by Correr and cols. and Novato and cols., respectively ( 9 , 10 ).

However, despite the existence of instruments already validated, the literature lacks evidence on the application and reporting of the results of these instruments. This may increase biases in the generation and interpretation of outcomes, as well as affect the use of this evidence for clinical practice.

In this context, the objective of our study was to systematically evaluate the application and reporting of the DQOL and DQOLY instruments in interventional and observational studies with type 1 or 2 DM patients. In addition, we discuss the relationships between QoL measured and clinical, demographic, psychological, and metabolic variables.

## MATERIALS AND METHODS

### Search and eligibility criteria

A systematic review was conducted according to the recommendations of the Cochrane Collaboration and PRISMA statement ( 11 , 12 ). All steps were conducted by two independent reviewers, with a third reviewer for consensus meetings. Systematic searches were conducted in the electronic databases Medline, Scopus, Web of Science, Scielo, and Lilacs. The searches were complemented by a manual search in the reference lists of included studies and in non-indexed records (Google Scholar). For the search, the following descriptors and their variants were used: diabetes, quality of life, DQOL, and DQOLY.

The acronym “PICOS” was used as an initial basis for establishing the eligibility criteria of the studies: P = patients (in any age group, sex, or ethnicity) with a diagnosis of DM (type 1 or 2) of any etiology, with or without comorbidities. We included studies evaluating any type of intervention (pharmacological or not) or only in follow-up of the patients (absence of intervention); I/C = application and/or evaluation of the use of DQOL and/or DQOLY tools; O = DQOL and/or DQOLY instruments measurements; S = interventional or observational studies.

We excluded studies with languages different from English, Portuguese, or Spanish; specific validation studies of instruments; instruments answered by third parties or not described; other types of instruments for QoL assessment; and patients with other etiologies of DM.

### Data extraction and summary of results

The studies found during the systematic search went through the screening stages (reading titles and abstracts), and the included studies were read in full and evaluated according to the eligibility criteria. The selected studies had their data extracted in pre-elaborated worksheets (Microsoft Excel). Data were extracted on (i) sociodemographic variables: age and sex; (ii) clinical variables: etiology of DM (type 1 or 2), interventions or follow-up, duration of disease (time from diagnosis), control and evolution of the disease (glycated hemoglobin – HbA1c%), presence of complications (neuropathy, hypertension, retinopathy, or nephropathy), and body mass index (BMI kg/m ^2^ ); and (iii) humanistic variables: QoL (score and evaluation in the DQOL or DQOLY instruments).

Regarding the DQOL and/or DQOLY tools, we evaluated (i) the instrument description (number of questions and domains) and adaptations; (ii) validation of the tool (Cronbach’s alpha, validated language); and (iii) interpretation of the results obtained.

The evidence was synthesized qualitatively and quantitatively. In the descriptive statistics, absolute and relative values or frequencies were used to describe categorical variables and trend measures for continuous variables.

Regarding the DQOL and/or DQOLY tools, we evaluated (i) the instrument description (number of questions and domains) and adaptations; (ii) score used (scoring scale); (iii) validation of the tool (Cronbach’s alpha, validated language); and (iv) interpretation of the results obtained.

The evidence was synthesized qualitatively and quantitatively. In the descriptive statistics, absolute and relative values or frequencies were used to describe categorical variables and trend measures for continuous variables.

## RESULTS

**Figure 1:**
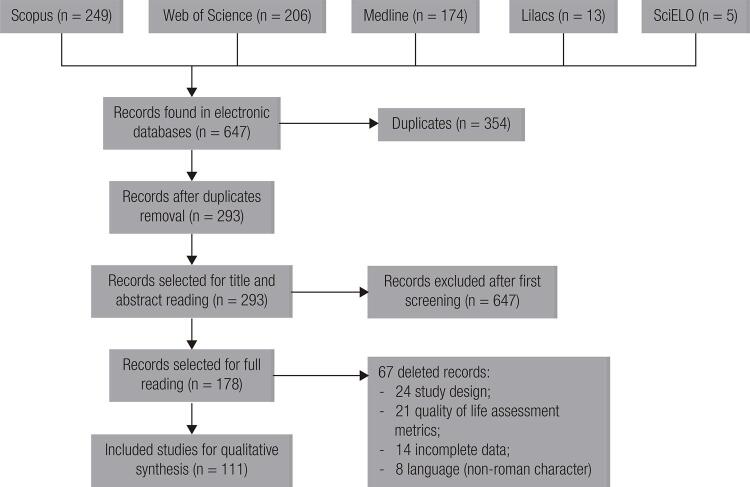
Flowchart of lhe study selection process

A total of 647 records were initially identified from the electronic databases. After the withdrawal of 354 duplicates, the titles and abstracts of 293 articles were screened, of which 178 were selected for reading in full. After this stage, 111 articles were eligible for qualitative evaluation, referring to 110 studies, including interventional (n = 32) and observational (n = 78) studies ( Figure 1 ). Table 1 presents the main characteristics of the included studies.

**Table 1:** Characteristics of included studies

	Interventional studies	Observational studies
Number of studies	32	78
Study design	20 (RCT) 9 (quasi-experimental) 2 (NR) 1 single-arm clinical study	50 (transversal) 16 (longitudinal) 10 (others) 2 (ambidirectional)
Evaluated interventions	12 (treatment efficacy) 9 (educational programs) 7 (behavioral) 4 (telemedicine)	Several (QoL assessment, follow-up, glycemic control, association with variables: gender, age)
Publication year	1988 to 2015 78.1% after 2000	1992 to 2016 92.3% after 2000
Country	9 USA – 28% 4 Canada – 12.5% 4 multicentric – 12.5%	25 USA – 32% 7 Brazil – 9% 7 Italy – 9%
Study duration	3 days to 6.5 years	4 months to 23.5 years*
Number of patients	6,612	20,869
Age	12 to 70 years	5.6 to 68.2 years
Male	50.7%	48.1%

* For longitudinal studies.

RCT: randomized controlled study; NR: not reported; QoL: quality of life.

Patients diagnosed with T1DM were evaluated in 69 studies and patients with T2DM in 35 studies. Six observational studies followed both types of patients. The mean time of DM ranged from two to 29 years, and the majority of the patients did not present the disease well controlled ( Table 2 ).

**Table 2:** Clinical characteristics of patients with DM included in the studies

	Interventional studies (N = 32)	Observational studies (N = 78)
DM duration (mean)	5.1 to 24 years	2 to 29 years
DM etiology	20 (T1DM) 12 (T2DM)	49 (T1DM) 23 (T2DM) 6 (both)
Basal HbA1c (mean)	6.1 to 11%	7 to 12.2%
Final HbA1c (mean)	5.9 to 9.5%	7.1 to 9.6% *(N = 14)
Other variables		BMI: 20 to 35 kg/m ^2^ (N = 37); Complications: neuropathy, nephropathy, or retinopathy (N = 26); Comorbidities: hypertension (N = 12)

N: number of studies; T1DM: type 1 diabetes mellitus; T2DM: type 2 diabetes mellitus; BMI: body mass index; HbA1c: glycated hemoglobin. * Studies that presented results for this variable.

In the interventional studies, the relationship of the QoL with the applied interventions was evaluated. Statistically significant improvement in QoL was observed in the patients in the intervention group compared to the control group in 13 studies (40.6%) ( Table 3 ).

**Table 3:** Interventions assessed in interventional studies

Interventions	N	IG > CG (p < 0.05)	Not significant	CG > IG (p < 0.05)	NR
Treatment efficacy	12	9	3	-	-
Educational programs	9	-	7	1	1
Behavioral	7	2	3	-	2
Telemedicine (mobile text message tracking, websites)	4	2	2	-	-

N: number of studies; IG: intervention group; CG: control group; NR: not reported.

Considering the use of instruments to assess QoL, 81 studies (73.6%) applied the DQOL instrument; 27 studies (24.5%) used the DQOLY; and two other trials (1.8%) used both tools. Modifications or adaptations of the DQOL and/or DQOLY tools were found in 11 studies, called DQOL-Brief, DQOL-Modified, and DQOLY-Short form.

**Figure 2:**
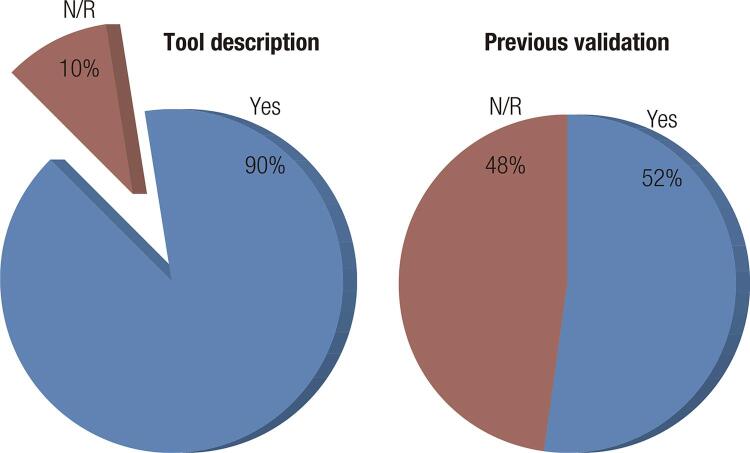
Data on instrument description and validation.

**Figure 3:**
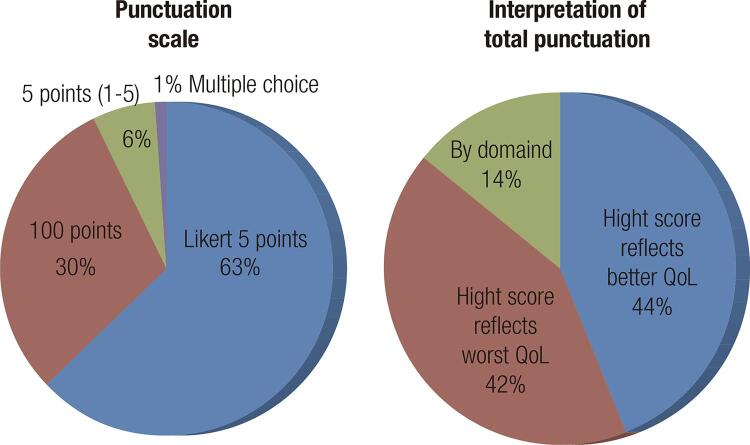
Punctuation and interpretation of DQOL and/or DQOLY data.

The information obtained in the studies with the application of the DQOL and/or DQOLY tools in relation to their description and validation parameters is summarized in Figure 2 . Regarding the instrument score, 77% of the studies reported this information. Figure 3 represents the instrument’s punctuation form (way of obtaining the points and scale used) and interpretation of the total scores obtained. Evidence was available on 110 studies.

## DISCUSSION

In the present study, the use of specific instruments already well established and validated in several countries, including Brazil, was evaluated for the QoL of patients with DM: DQOL and DQOLY. Both interventional and observational studies were systematically evaluated to obtain an overview of the application and reporting of these instruments. The shorter version of the instrument makes it easier to apply in clinical practice, since the modified version allows adaptation to the peculiarities of the study and the study population ( 13 , 14 ).

These instruments are useful not only to know the reality of the patient at a given moment of time, but also to determine changes in QoL after an intervention, be they educational, psychological, therapeutic or pharmacological, or follow-up to treatment ( 15 ).

Although the vast majority of studies (90%) described the QoL tool used, only half reported the validation or language of the instrument in the study. This creates an impasse, because it makes it difficult to evaluate the quality of the decisions or evidence (validity) and the quality of the data obtained (reliability) ( 16 ). In terms of instrument scores, most studies used a five-point Likert scale. This is probably because this type of scale is widely used and easy to interpret. The Likert scale measures attitudes and behaviors using response options that vary from one extreme to the other (very satisfied to very dissatisfied) and allows to discover levels of opinion ( 17 ). In the original instrument, the score is made by a Likert scale of five points, ranging from 1 (very satisfied or slightly worried) to 5 (unsatisfied or very worried) ( 7 ).

Regarding the interpretation of the DQOL and/or DQOLY data, it was observed that the studies do not follow a standard. The authors who describe the interpretation of the results do so in directly proportional scales in half of the studies, while in the other half the results are evaluated in inversely proportional scales. In addition, some studies present the interpretation of the results by domains. This is probably due to the differences inherent in the validations, translations, and adaptations of the instruments in different countries according to the local population and cultures in order to increase the specificity and precision of the measurements of the QoL ( 18 ). However, greater standardization would have to exist in order to avoid bias and misinterpretation.

The overall results of the studies evaluated in the systematic review demonstrated that sociodemographic and cultural variables may be correlated with QoL, as already mentioned in a study by Kiadaliri and cols. ( 19 ). In this study, the authors reported low QoL results associated mainly with Iranian patients (women) with worse disease control (higher HbA 1 c values) and advanced age with higher complications and lower socioeconomic level ( 19 ). The study by Trento and cols. also shows that the QoL of patients with T1DM is influenced by the age of the patient at the time of diagnosis of the disease, and patients of advanced age have a worse perception of QoL. This is probably due to the earlier diagnosis and care of DM incorporated as part of the routine and, consequently, better acceptance of the pathology and its treatment, with a lower impact on the patient’s life ( 20 ). Regarding the gender variable, other studies report differences in the perception of QoL, with women generally presenting scores that reflect a worse perception of QoL compared to men ( 21 ).

In addition to these aspects, we highlight the clinical variables as affecting the QoL of patients with DM. Several studies have shown that patients with better metabolic control (considering the reduction in HbA1c levels) have a better QoL ( 3 ). This is mainly related to better control of the disease, which reduces DM complications ( 22 ). In this review, baseline mean HbA1c ranged from 6.1% to 11% in interventional studies and from 7% to 12.2% in observational studies. It was reduced in the range of 5.9% to 9.5% in interventional studies and 7.1% to 9.6% in observational studies after the interventions or follow-up of the patients. Even with this reduction, these values reflect, in accordance with the DM guidelines, poor metabolic control of the disease and may have a substantial impact on the results obtained for QoL in these studies ( 4 , 23 ).

A review conducted by Smith-Palmer and cols. ( 24 ) showed that both acute and chronic complications of DM are associated with worsening of QoL indexes. The etiology of DM may reflect patients’ QoL, and, in general, T1DM patients report better QoL than those with T2DM, probably due to the younger age of the T1DM group and the reduced presence of complications of the disease in this group ( 25 ). Chronic complications most associated with worse QoL include stroke and blindness ( 24 ). Other studies also mention neuropathy, nephropathy, and retinopathy as the main causes of QoL reduction. In addition, the number of complications and the severity of these complications are strong predictors of QoL in diabetic patients ( 26 ). In the present systematic review, the main complications were retinopathy, neuropathy, and nephropathy. Only a third of the studies mention this type of subanalysis, because most of the studies have as one of the exclusion criteria the withdrawal of diabetic patients with complications, which makes a more in-depth analysis impossible. In observational studies such as cohorts, it would be important to include patients with this type of profile who better reflect real-world settings.

Other modifiable risk factors such as BMI and hypertension may also influence QoL in diabetic patients, although these factors have been evaluated in a small number of studies – as noted in our systematic review – and should be better investigated ( 24 ).

From the included observational studies, some (transversal and longitudinal cohort studies) investigated certain psychological aspects of the patients, correlating QoL and depression/anxiety in young people with DM. The results demonstrate a direct relationship between these variables, indicating that the presence of depression and anxiety in the patients reduces the perception of QoL ( 27 ). The review conducted by Naskar and cols. ( 28 ) also shows the relationship between depression and DM in Indian patients, concluding that QoL is strongly related to psychological aspects in chronic diseases, which is a common comorbidity mainly in patients with T2DM. Other studies have shown a higher prevalence of depression in subjects with longer duration of diabetes (≥ 5 years), poorer glycemic control (HbA1c > 7%), and patients using insulin ( 28 , 29 ). Similar data were found in this review.

Drug treatment (efficacy) was evaluated in most of the included interventional studies, and insulin therapy (intensive treatment) was the most studied because of the negative impact on QoL. Since the publication by the DCCT of the importance of intensive treatment for diabetes control, many studies have evaluated the impact of these strategies on individuals’ daily life. The study from Ridderstrale and cols. shows that the simpler treatment regime, with fewer daily injections and less planning, has a positive impact on QoL ( 3 ).

Another important factor evaluated in the interventional studies was the relationship of treatment (insulin vs. oral antidiabetics) in patients’ QoL. Studies report an improvement in glycemic control with the use of insulin, but worsening in patients’ QoL ( 30 ). In addition, studies report an improvement in QoL (satisfaction with treatment) and symptoms of hypoglycemia in patients using an insulin infusion pump compared to those using multiple doses of insulin (three to four times a day) ( 31 ). The use of devices for continuous infusion of insulin was studied as an alternative to improve adherence to treatment and metabolic control. Some cross-sectional, longitudinal, and comparative observational studies have demonstrated that the use of the device is able to improve metabolic control and positively reflect QoL ( 32 ).

An important aspect to be considered regarding the choice of drug treatment is the possibility of opposition in the use of insulin in patients with T2DM. This is because hypoglycemia and weight gain were cited as barriers to the use of insulin treatment, negatively impacting QoL and adherence to treatment ( 30 ). In addition, research has shown that the best results of glycemic control and QoL are obtained by empowering the patient for decision-making (self-monitoring) through educational programs and the follow-up of a multiprofessional team ( 33 ).

Regarding the interventional studies that analyze the effect of other types of interventions besides drugs, no statistically significant differences were found in the QoL. The following were evaluated: the application of an educational program (guidelines on pathology, self-monitoring, patient empowerment for decision-making), telemedicine (supported by technology: websites, mobile messages, telephone contact), and behavioral intervention (family, marital, and work support) ( 34 , 35 ).

In general, the evaluation of interventional studies allowed us to obtain an overview of the main interventions studied up to the present moment in DM, as well as their impact on the QoL. However, long-term health effects and outcomes considering the patient’s actual (non-randomized clinical) scenario are generally not assessed in this type of study. On the other hand, observational studies allowed us to visualize other aspects of QoL in patients with DM, mainly related to sociodemographic and psychological aspects and disease control. However, the high heterogeneity of these studies did not allow for more comparisons and quantitative analyses.

Finally, due to the complexity of DM, several dimensions need to be considered for the definition of the treatments (pharmacological and non-pharmacological) and patient follow-up, including disease etiology, comorbidities, and sociodemographic aspects, since these also have a great impact on patients’ QoL. The use of standardized instruments for QoL assessment is essential for generating more consistent evidence. In addition, it is strongly recommended that the authors standardize the application of the instruments and reporting of results. Reviewers and editors should require that information on the validation and punctuation of such instruments be provided to enable greater transparency and reproducibility of evidence.

In conclusion, the use of the DQOL and DQOLY instruments shows different results due to the application, validations, adaptations, and translations of the tools, as well as the intrinsic subjectivity of these instruments, evaluated populations, and types of studies. This fact may present an obstacle in gathering health information and make it difficult to interpret the results. The application of the tools in clinical practice requires an appropriate and accurate understanding of the instruments and of psychometric and statistical concepts, and their application in studies should be standardized.

Overall, the studies show a high impact of DM on patients’ QoL, with the main correlated variables of age, sex, disease diagnosis time, glycemic control, and presence of complications/comorbidities. More studies considering patients in real-world settings are needed to better elucidate these correlations.
